# Increased risks between Interleukin-10 gene polymorphisms and haplotype and head and neck cancer: a meta-analysis

**DOI:** 10.1038/srep17149

**Published:** 2015-11-27

**Authors:** Yu-Ming Niu, Xin-Ya Du, Heng-Xing Cai, Chao Zhang, Rui-Xia Yuan, Xian-Tao Zeng, Jie Luo

**Affiliations:** 1 Department of Stomatology, Taihe Hospital, Hubei University of Medicine, 32 South Renmin Road, Shiyan 442000, P.R. China; 2 Department of Stomatology, People’s Hospital of New District Longhua Shenzhen, Shenzhen 518109, P.R. China; 3 The State Key Laboratory Breeding Base of Basic Science of Stomatology & Key Laboratory of Oral Biomedicine, Ministry of Education, Department of Oral and Maxillofacial Surgery, School & Hospital of Stomatology, Wuhan University, Luoyu Road 237, Wuhan 430079, P.R. China; 4 Center for Evidence-Based Medicine and Clinical Research, Taihe Hospital, Hubei University of Medicine, 32 South Renmin Road, Shiyan 442000, P.R. China; 5 Center for Evidence-Based and Translational Medicine, Zhongnan Hospital, Wuhan University, 169 Donghu Road, Wuchang District, Wuhan, 430071, P.R. China

## Abstract

Molecular epidemiological research suggests that interleukin-10 (IL-10) polymorphisms may be associated with an increased risk of head and neck cancer (HNC), but results remain controversial. To derive a more precise evaluation, we performed a meta-analysis focused on genetic polymorphisms of IL-10. PubMed, Embase, CNKI and Wanfang databases were searched for studies that examined the relationship between IL-10 polymorphisms or haplotypes and HNC risk. The odds ratio (OR) and 95% confidence interval (CI) were applied to assess the relationship strength. Publication bias, sensitivity and cumulative analyses were conducted to measure the robustness of our findings. Overall, nine related studies involving 2,258 patients and 2,887 control samples were analyzed. Significant associations between the IL-10-1082A > G polymorphism and HNC risk were observed (G vs. A: OR = 1.56, 95% CI = 1.27–1.92, P < 0.01, I^2^ = 69.4%; AG vs. AA: OR = 1.64, 95% CI = 1.32–2.05, P < 0.01, I^2^ = 55.6%; GG vs. AA: OR = 2.24, 95% CI = 1.69–2.97, P < 0.01, I^2^ = 38.5%; AG + GG vs. AA: OR = 1.70, 95% CI = 1.36−2.14, P = 0.02, I^2^ = 61.8%; GG vs. AA + AG: OR = 1.89, 95% CI = 1.23−2.90, P = 0.01, I^2^ = 46.3%) in the total population, as well as in subgroup analysis. Moreover, increased HNC risks were also associated with the IL-10 −819T > C polymorphism and the GCC haplotype. In conclusion, our meta-analyses suggest that IL-10 polymorphisms, specifically the −1082A > G polymorphism, may be associated with increased risk of HNC development.

Head and neck cancer (HNC) is one of the most common malignancies and derives from the malignant transformation of the epithelial cells that line the upper respiratory tract and digestive system^1^,^2^. HNC accounts for a large proportion of cancer-related deaths and constitutes approximately 3.31% of all cancers in the United States, with 55,070 new cases and 12,000 deaths in 2014^3^. Significant improvement in the 5-year disease-specific survival (DSS) was achieved due to recent advances in treatment, from 55% in 1992–1996 to 66% in 2002–2006^4^. The development of prophylactic strategies remains critical for managing HNC. For example, many studies have demonstrated that HNC is associated with complex environmental factors, such as tobacco use, alcohol consumption, and intake of vitamins and minerals. In addition, infection with human papillomavirus (HPV) and dental trauma are also thought to be potential HNC risk factors^5^,^6^,^7^,^8^,^9^,^10^.

Interestingly, these factors do not explain the variation in susceptibility observed within different populations. Over the past decade, many studies have suggested that leukocytes and their relevant cytokines may play a central role in inflammatory infiltration and malignant transformation^11^,^12^,^13^. Interleukin-10 (IL-10) is a cytokine produced by monocytes, macrophages, lymphocytes and other human Thl cells^14^, that plays a functional role in inflammatory and immunoregulatory activities^15^. During carcinogenesis, IL-10 functions in both immune suppression (cancer promotion) and anti-angiogenesis (cancer inhibition)^16^.

IL-10 is an important inflammatory cytokine with anti-inflammatory properties. The IL-10 gene is located on chromosome 1 at q31-32, contains five exons and four introns that span a length of 4.8 kb and encode a 178-amino-acid protein^17^,^18^,^19^. Mutations in IL-10 have been detected at several loci, and the association between IL-10 SNPs and disease risk has been heavily studied. The three most common IL-10 SNPs are located in the promoter region (−1082A > G (rs1800870), −819T > C (rs1800871) and −592A > C (rs1800872)) and have been reported to regulate IL-10 transcription and expression^20^,^21^,^22^. These mutations influence IL-10 gene transcription and translation, resulting in abnormal cell proliferation and cancer development^23^,^24^. Numerous molecular epidemiological studies have investigated the association betweenIL-10 gene polymorphisms and cancer risk, such as breast cancers^25^, lung cancer^26^, cervical cancer^27^, and digestive cancer^28^. In 2006, Pratesi *et al.*^29^ published the first study describing the negative association between three IL-10 promoter polymorphisms and the risk of nasopharyngeal cancer in Italian populations. However, the results were not consistent with subsequent studies. To date, no published genome-wide association studies (GWAS) have been performed to explore the precise association between IL-10 polymorphisms and HNC risk. Given the important role of IL-10 in the development of HNC cancer, we conducted a meta-analysis to assess the relationship between IL-10 polymorphisms (−1082A > G, −819T > C and −592A > C) and HNC risk.

## Methods

This meta-analysis was designed according to the guidelines described in the Preferred Reporting Items for Systematic Reviews and Meta-analyses (PRISMA Compliant) statement^30^.

### Search strategy

The Pubmed, Embase CNKI and Wanfang databases were searched to identify studies that examined the association between HNC and IL-10 polymorphisms, using the following search terms: “head and neck cancer”, “oral cancer”, “nasopharyngeal cancer”, “pharynx cancer”, “larynx cancer”, “Interleukin-10”, “IL-10”, “polymorphism”, and “variant”.

### Eligible criteria

All selected studies met the following criteria: 1) the research design was a case-control study; 2) the study focused on the association between the IL-10 polymorphisms and HNC susceptibility; 3) the study included sufficient genotype distribution data to calculate odds ratios (ORs) and 95% confidence intervals (CIs); 4) the study was published in either Chinese or English. The largest sample size or most recent publication was preferentially selected in cases of overlapping data or duplicate publications.

### Data extraction

The following information was extracted from all qualified studies by two independent researchers (Niu and Cai): first author, publication year, country, racial descent (categorized as either Asian or Caucasian), source of controls, number of cases and controls with different genotypes, genotyping method, Hardy-Weinberg equilibrium (HWE) for controls, cancer location and study quality assessment.

### Quality assessment

Two independent authors (Niu and Du) assessed the quality of the included studies using quality scoring criteria modified from previous meta-analyses (Supplemental Table S1 online, which demonstrated the scale for quality assessment.)^31^,^32^. Modified criteria were based on traditional quality scoring used for observational studies in genetic epidemiological issues and ranged from 0 points (worst) to 9 points (best). Studies with a score of 6 or higher were classified as high quality, whereas studies with a score of 6 or less were classified as low quality.

### Statistical analysis

ORs with 95% CIs were used to assess the strength of the association between the IL-10-1082A > G, −819T > C and −592A > C polymorphisms and HNC risk. For the IL-10–1082A > G polymorphism, pooled ORs were obtained for allele contrast (G vs. A), co-dominant model (AG vs. AA; GG vs. AA), dominant model (GA + GG vs. AA), and recessive model (GG vs. AA + GA). These similar genetic models were also used to assess the IL-10 −819T > C and −592A > C polymorphisms. Subgroup analyses were performed based on cancer location, HWE status of controls, ethnicity, and study design. ORs were calculated using the random-effects model (DerSimonian and Laird method) when the P-value was less than 0.10 or I^2^ was greater than 40%^33^. Otherwise, a fixed-effect model (the Mantel-Haenszel method) was adopted^34^. Cumulative meta-analyses and sensitivity analysis were conducted to evaluate the overall robustness of the study’s results. Publication bias was analyzed using Egger’s linear regression and Begg’s funnel plots^35^. In addition, heterogeneity was assessed using Cochran’s Q statistic and the I^2^ method, and meta-regression was conducted to analyze heterogeneity^36^. Statistical analysis was performed using STATA version 11.0 (Stata Corporation, College Station, TX, USA) with a two-sided P-value, P < 0.05 was considered significant.

## Results

### Study characteristics

**Figure 1:**
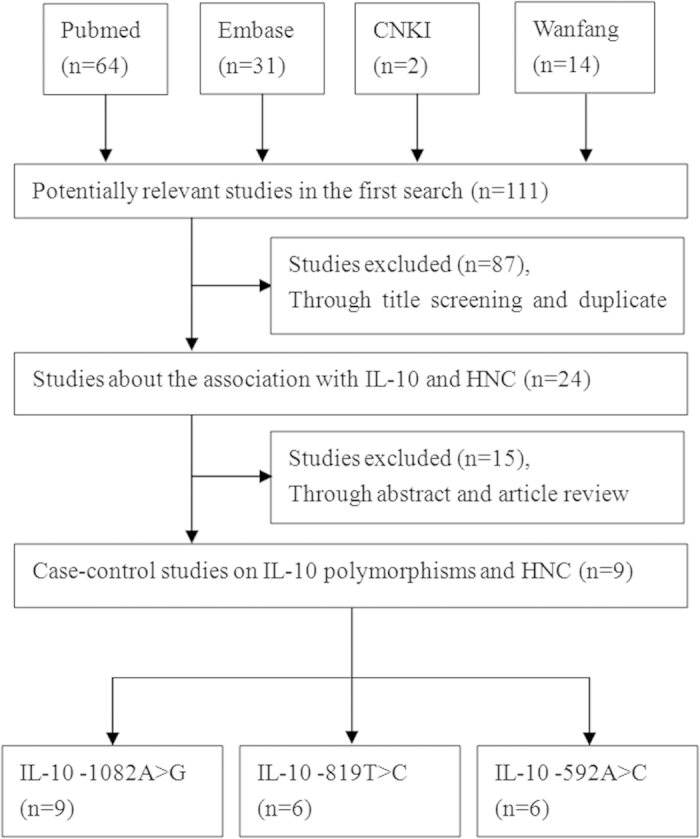
Flow diagram of the study selection process.

A systematic review of the literature identified 111 relevant studies. Figure 1 and PRISMA flow diagram (Supplemental Table S2 and S3 online) show a flow chart of the studies selection procedure. One hundred and two studies were excluded. Ultimately, nine studies^29^,^37^,^38^,^39^,^40^,^41^,^42^,^43^,^44^ satisfied the outlined inclusion criteria and characteristics (Table 1 and Supplemental Table S4 online), including the quality assessment score that was generated based on the Newcastle-Ottawa Scale of studies. Each of the nine studies focused on the association of the −1082A > G polymorphism with HNC risk^29^,^37^,^38^,^39^,^40^,^41^,^42^,^43^,^44^, and six studies investigated the association of the −819T > C and −592A > C variants with HNC risk^29^,^37^,^39^,^42^,^43^,^44^. Three studies focused on Caucasian populations^29^,^38^,^40^, and six studies focused on Asian populations^37^,^39^,^41^,^42^,^43^,^44^. Within the distribution of genotypes in the control groups, only three studies exhibited HWE in the −1082A > G polymorphism^29^,^41^,^44^, and only one study deviated from HWE in the −819T > C and −592A > Cvariants^42^.

**Figure 2:**
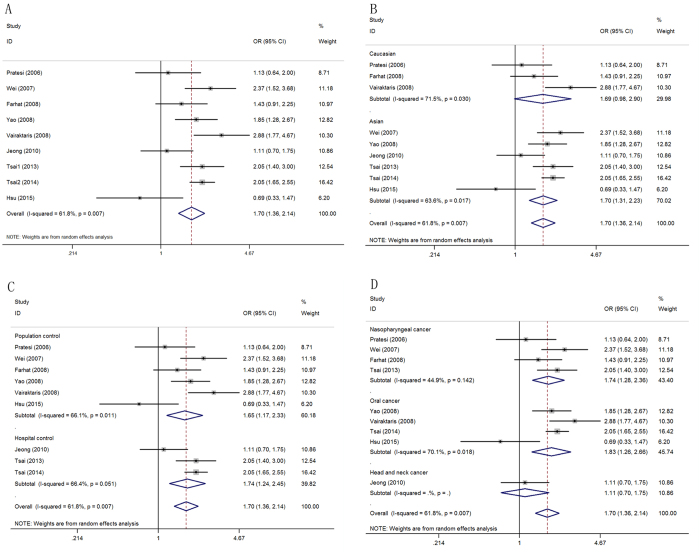
Calculated OR and 95% CIs for the associations between IL-10 −1082A > G polymorphism and HNC risk in the AG + GG vs. AA model ((A) for overall populations; (B) for ethnicity subgroup; (C) for control sources subgroup; (D) for cancer location subgroup).

Association between the IL-10 −1082A > G polymorphism and HNC risk A total of 9 relevant studies, consisting of 2,258 patients and 2,887 controls, were examined for the association between the IL-10 −1082A > G polymorphism and HNC risk. The combined analyses revealed a significantly increased risk of HNC risk for this mutation in all five genetic models (G vs. A: OR = 1.56, 95% CI = 1.27–1.92, P < 0.01, I^2^ = 69.4%; AG vs. AA: OR = 1.64, 95% CI = 1.32–2.05, P < 0.01, I^2^ = 55.6%; GG vs. AA: OR = 2.24, 95% CI = 1.69–2.97, P < 0.01, I^2^ = 38.5%; AG + GG vs. AA: OR = 1.70, 95% CI = 1.36–2.14, P = 0.02, I^2^ = 61.8%, Fig. 2; GG vs. AA + AG: OR = 1.89, 95% CI = 1.23–2.90, P = 0.01, I^2^ = 46.3%; Table 2). Subsequent analyses accounting for ethnicity revealed similar results in Asian populations, using all five genotype models. Enhanced HNC risk was also observed in Caucasians for the AG vs. AA model and the dominant model. Significant correlations with increased HNC risk were also observed with all five genetic models in the hospital control group and four genetic models (except for the recessive model) in population-based control groups. Moreover, elevated risks of oral cancer (G vs. A: OR = 1.76, 95% CI = 1.36–2.27, P < 0.01, I^2^ = 55.2%; AG vs. AA: OR = 1.71, 95% CI = 1.15–2.54, P = 0.01, I^2^ = 71.1%; GG vs. AA: OR = 3.13, 95% CI = 2.06–4.77, P < 0.01, I^2^ = 0%; AG + GG vs. AA: OR = 1.83, 95% CI = 1.26–2.66, P < 0.01, I^2^ = 70.1%; GG vs. AA + AG: OR = 2.69, 95% CI = 1.77–4.09, P < 0.01, I^2^ = 0%) and nasopharyngeal cancer (G vs. A: OR = 1.53, 95% CI = 1.06–2.20, P = 0.02, I^2^ = 76.5%; AG vs. AA: OR = 1.75, 95% CI = 1.38–2.21, P < 0.01, I^2^ = 5.2%; AG + GG vs. AA: OR = 1.74, 95% CI = 1.28–2.36, P = 0.02, I^2^ = 44.9%; Table 2) were detected.

Heterogeneity was observed in the following four models: G vs. A, AG vs. AA, AG + GG vs. AA and GG vs. AA + AG. Meta-regression analyses highlighted ethnicity as a major driver of heterogeneity in the G vs. A model (τ2 = 100%, P = 9 × 10^−3^) and HWE deviation responsible for other models (AG vs. AA: τ2 = 100%, P = 6 × 10^−3^; AG + GG vs. AA: τ2 = 100%, P = 9 × 10^−3^; and GG vs. AA + AG: τ2 = 70.12%, P = 0.02). Importantly, heterogeneity was relieved in the subgroup analysis.

**Figure 3:**
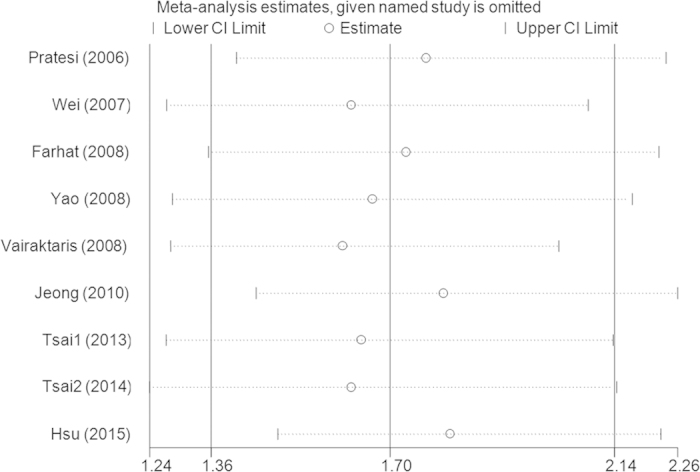
Sensitivity analysis via deletion of each individual study reflects the relative influence of each individual dataset on the pooled ORs in the AG + GG vs. AA model ofIL-10 −1082A > G polymorphism.

**Figure 4:**
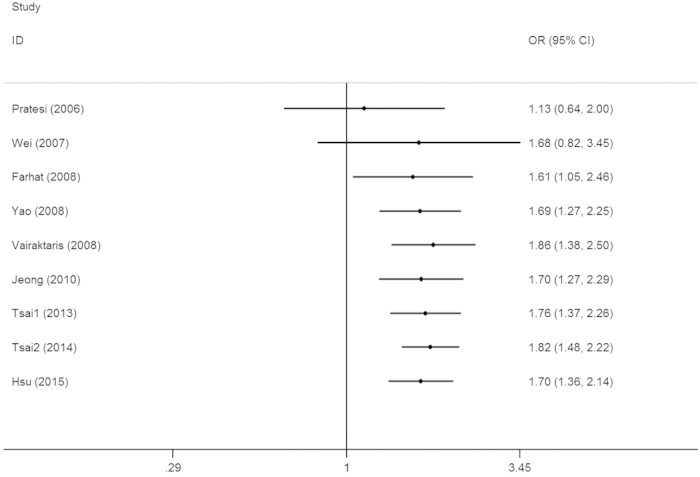
Cumulative meta-analyses according to publication year in the AG + GG vs. AA model of IL-10 −1082A > G polymorphism.

Sensitivity analysis revealed that no single study qualitatively changed the pooled ORs, indicating that the results of this meta-analysis were stable (Fig. 3 for AG + GG vs. AA model). Cumulative analysis by publication date demonstrated that cancer risk increased gradually and became positive following the study conducted by Farhat *et al.* in 2008 (Fig. 4 for AG + GG vs. AA model).

**Figure 5:**
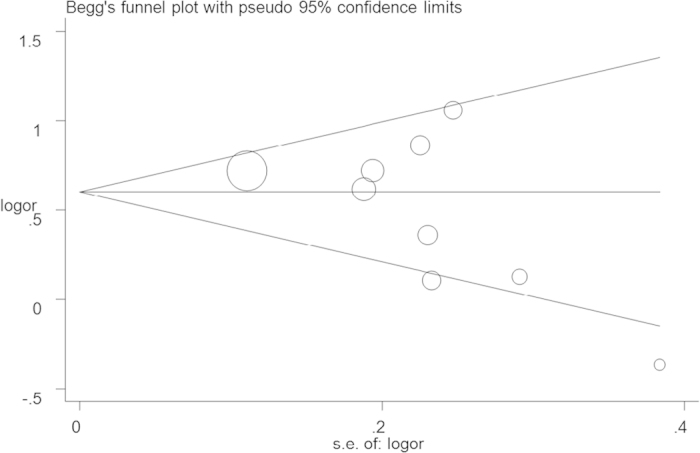
Funnel plot analysis to detect publication bias for AG + GG vs. AA model of IL-10 −1082A > G polymorphism. Circles represent the weight of the studies.

Funnel plots were conducted to assess the publication bias, and no evidence of asymmetry was observed (Fig. 5 for AG + GG vs. AA model). This result was further supported by the analysis using Egger’s test (G vs. A: P = 0.09; AG vs. AA: P = 0.17; GG vs. AA: P = 0.69; AG + GG vs. AA: P = 0.12; GG vs. AA + AG: P = 0.57).

Association between the IL-10 −819T > C polymorphism and HNC risk Six studies consisting of 1,676 cases and 2,230 controls were included in the analysis to determine whether the IL-10 −819T > C polymorphism was associated with HNC risk. A significant increase in HNC risk was observed in the overall population (C vs. T, OR = 1.15, 95% CI = 1.04–1.21, P = 0.01, I^2^ = 20.1%; CC vs. TT, OR = 1.28, 95% CI = 1.03–1.59, P = 0.03, I^2^ = 0%), as well as among Asian populations (C vs. T, OR = 1.16, 95% CI = 1.05–1.28, P < 0.01, I^2^ = 29.7%; CC vs. TT, OR = 1.30, 95% CI = 1.04–1.61, P = 0.02, I^2^ = 0%; Table 2). Furthermore, analysis restricted to HWE studies revealed elevated cancer risk in homozygous comparison (OR = 1.24, 95% CI = 1.07–1.45, P = 0.01, I^2^ = 33.3%) and recessive model (OR = 1.53, 95% CI = 1.08–2.15, P = 0.02, I^2^ = 0%). Moreover, these results were consistent with subgroup analysis of the population control group (Table 2). Sensitivity analysis and cumulative analysis were conducted, and no conspicuous change of the pooled ORs was detected. No publication bias was observed, indicating that the results are statistically robust (C vs. T: P = 0.32; TC vs. TT: P = 0.64; CC vs. TT: P = 0.82; TC + CC vs.TT: P = 0.82; CC vs. TT + TC: P = 0.71).

Association between the IL-10 −592A > C polymorphism and HNC risk Six studies consisting of 1,676 cases and 2,230 controls were included in the analysis to determine whether the IL-10–592A > C polymorphism was associated with HNC risk. Overall, no significant association was observed in all five models (Table 2). Only two genetic models (for CC vs. AA, OR = 1.24, 95% CI = 1.07–1.45, P = 0.01, I^2^ = 33.3%; for CC vs. AA + AC, OR = 1.53, 95% CI = 1.08–2.15, P = 0.02, I^2^ = 0%) revealed increased risk of HNC in the population control group. Further subgroup analysis of ethnicity and cancer type was conducted, and no significant association was identified. The pooled ORs did not exhibit any change with sensitivity and cumulative analysis, and no publication bias was observed (C vs. A: P = 0.26; AC vs. AA: P = 0.45; CC vs. AA: P = 0.43; AC + CC vs. AA: P = 0.85; CC vs. AA + AC: P = 0.52).

Association between the IL-10 haplotype and HNC risk Of the selected studies, only four described the association between the IL-10 −1082A > G, −819T > C and −592A > C haplotype and HNC risk (Table 3). Quantitative synthesis indicated that only the GCC haplotype was associated with a significant increase in HNC risk in the overall population (GCC vs. ATA, OR = 1.44, 95% CI = 1.04–2.00, P = 0.03, I^2^ = 52.6%; Table 4).

## Discussion

Genetic factors have been shown to influence the susceptibility of patients to various diseases and have attracted increasing attention^45^,^46^. Inflammation and immune cytokines play an important role during the malignant progression of normal epithelium to cancer by driving angiogenesis, cell metastasis and immune-suppression^47^,^48^,^49^. Genetic mutations influence the transcription and translation of genes, resulting in abnormal expression of corresponding mRNAs and proteins and deregulation of various cellular processes, such as apoptosis and proliferation.

To date, numerous molecular epidemiological studies have been conducted to evaluate the association between polymorphisms of IL-10 and the risk of HNC development, but results have remained conflicting. Regarding the IL-10 −1082A > G polymorphism, Wei *et al.* reported that the AG and GG genotypes were associated with a significant increase in HNC risk compared with the AA genotype in a Chinese population (95% CI = 1.37–3.48 and 1.33–10.84)^37^. A similar increase in HNC risk was also observed in other studies that focused on different ethnicities^39^,^40^,^42^,^43^, whereas other studies identified no significant association between the IL-10 −1082A > G polymorphism and HNC risk^29^,^38^,^41^.

In 2014, a meta-analysis that included four studies focused on nasopharyngeal cancer was published and reported an increased risk with the IL-10 −1082A > G polymorphism^50^. To our knowledge, this is the first and most comprehensive meta-analysis to date that has explored the association between the IL-10 polymorphisms and HNC risk, more relative studies of HNC and more polymorphisms of IL-10 are collected to make a precise conclusion. Our meta-analysis includes nine studies, consisting of 2,258 patients and 2,887controls, and revealed that the IL-10-1082A > G polymorphism is associated with a significant increased risk of HNC. In subgroup analyses by ethnicity, we found that individuals with the G allele and mutated genotypes had a significant HNC risk compared with healthy individuals in Asian populations, suggesting that the increased HNC risk may be ethno-specific. Six case-control studies of IL-10 −819T > C and−592A > C polymorphisms were examined, including 1,676 cases and 2,230 controls. Positive correlations were observed in the −819T > C polymorphism analyses but not for the −592 A > C polymorphism.

In the current analysis, we found that the frequency of GCC haplotype was higher in the HNC patients than in controls and was associated with a significant increased HNC risk. The GCC haplotype of the IL-10 promoter may result in increased transcription and IL-10 expression. These effects down-regulate the expression of Th1 cytokines, thus allowing tumor cells to escape immune surveillance^37^. However, due to the small sample size and the limited number of studies examined, it remains unclear whether the GCC haplotype is indeed a marker of HNC patients. Further research is warranted to investigate this relationship.

Stratified analysis was successfully used to relieve moderate heterogeneity bias in the IL-10–1082A > G polymorphism analysis within the Asian population and the oral cancer group, suggesting that ethnicity and cancer location may influence heterogeneity. Moreover, we did not observe any publication bias in the three polymorphisms, demonstrating that the results of this meta-analysis are stable.

It is important to note the limitations of our meta-analysis. First, all of our results may be influenced by casualness due to the small number of studies included and the limited sample size of each study. Larger sample sizes are necessary to accurately clarify the association between the IL-10 polymorphisms and HNC risk. Second, only articles published in English or Chinese were selected, potentially causing a language bias. Third, HNC is a multi-factorial malignant tumor that likely arises from complex interactions between genetic mutations, environmental changes, lifestyle, diet, age and gender. Meta-analysis is a retrospective approach^51^, and the fundamental underlying mechanisms cannot be explained clearly due to unadjusted databases.

## Conclusion

Despite these limitations, our results suggest that the IL-10 −1082A > G polymorphism is a risk factor for HNC, especially in Asian populations. Our findings also indicate that the IL-10 −819T > C polymorphism also plays an important role in HNC development. No significant association was detected between the IL-10 −592A > C polymorphism and HNC risk. Moreover, the GCC haplotype was associated with an increased risk of HNC. In the future, additional studies with larger sample sizes are needed to identify the precise pathogenesis of IL-10 polymorphisms in HNC.

## Additional Information

**How to cite this article**: Niu, Y.-M. *et al.* Increased risks between Interleukin-10 gene polymorphisms and haplotype and head and neck cancer: a meta-analysis. *Sci. Rep.* **5**, 17149; doi: 10.1038/srep17149 (2015).

## Supplementary Material

Supplementary Information

## Figures and Tables

**Table 1:** Characteristics of case-control studies on IL-10 −1082A > G and −819T > C and −592A > C polymorphisms and cancer risk included in the meta-analysis.

Firstauthor	Year	Country	Racialdescent	Source of controls	Case	Control	Genotype distribution						*P*forHWE^a^	Genotypingmethod	CancerLocation	Qualityscore
							Case			Control						
							A/A	A/G	G/G	A/A	A/G	G/G				
Pratesi	2006	Italy	Caucasian	Population-control	89	130	29	41	19	46	58	26	0.33	Direct sequencing	Nasopharyngeal	7
Wei	2007	China	Asian	Population-control	198	210	123	61	14	167	38	5	0.01	PCR-RFLP	Nasopharyngeal	6
Farhat	2008	Qatar	Caucasian	Population-control	160	156	58	80	22	70	60	26	0.04	AS-PCR	Nasopharyngeal	6
Yao	2008	China	Asian	Population-control	280	300	184	75	21	234	56	10	0.01	PCR–RFLP	Oral	6
Vairaktaris	2008	Greek/Germany	Caucasian	Population-control	144	141	46	96	2	81	60	0	<0.01	PCR–RFLP	Oral	6
Jeong	2010	Korean	Asian	Hospital-control	278	350	238	38	2	304	45	1	0.62	TaqMan	HN	6
Tsai1	2013	China	Asian	Hospital-control	176	522	117	49	10	419	92	11	0.03	PCR–RFLP	Nasopharyngeal	6
Tsai2	2014	China	Asian	Hospital-control	788	956	522	217	49	766	168	22	<0.01	PCR–RFLP	Oral	6
Hsu	2015	China	Asian	Population-control	145	112	130	14	1	96	16	0	0.42	PCR-SSP	Oral	8
							T/T	T/C	C/C	T/T	T/C	C/C				
Pratesi	2006	Italy	Caucasian	Population-control	89	130	5	36	48	6	54	70	0.27	Direct sequencing	Nasopharyngeal	7
Wei	2007	China	Asian	Population-control	198	210	82	81	35	94	92	24	0.84	PCR-RFLP	Nasopharyngeal	7
Yao	2008	China	Asian	Population-control	280	300	113	120	47	129	134	37	0.66	PCR–RFLP	Oral	7
Tsai1	2013	China	Asian	Hospital-control	176	522	88	69	19	285	185	52	0.01	PCR–RFLP	Nasopharyngeal	6
Tsai2	2014	China	Asian	Hospital-control	788	956	418	288	82	528	335	93	<0.01	PCR–RFLP	Oral	6
Hsu	2015	China	Asian	Population-control	145	112	33	101	11	53	51	8	0.36	PCR-SSP	Oral	8
							A/A	A/C	C/C	A/A	A/C	C/C				
Pratesi	2006	Italy	Caucasian	Population-control	89	130	5	36	48	6	54	70	0.27	Direct sequencing	Nasopharyngeal	7
Wei	2007	China	Asian	Population-control	198	210	82	81	35	94	92	24	0.84	PCR-RFLP	Nasopharyngeal	7
Yao	2008	China	Asian	Population-control	280	300	113	120	47	129	134	37	0.66	PCR–RFLP	Oral	7
Tsai1	2013	China	Asian	Hospital-control	176	522	93	66	17	261	205	56	0.10	PCR–RFLP	Nasopharyngeal	7
Tsai2	2014	China	Asian	Hospital-control	788	956	408	301	79	484	374	98	0.04	PCR–RFLP	Oral	6
Hsu	2015	China	Asian	Population-control	145	112	33	101	11	53	51	8	0.36	PCR-SSP	Oral	8

MAF: Minor allele frequency in control group.

HN: All cancer locates in head and neck region, no specific description in original article.

^a^HWE in control.

**Table 2:** Summary ORs and 95% CI of IL-10 −1082A > G and −819T > C and −592A > C polymorphisms and head and neck cancer risk.

−1082 A>G	N*	G vs. A				AG vs. AA				GG vs. AA				AG + GG vs. AA				GG vs. AA + AG				
		OR	95% CI	*P*	*I*^*2*^	OR	95% CI	*P*	*I*^*2*^	OR	95% CI	*P*	*I*^*2*^	OR	95% CI	*P*	*I*^*2*^	OR	95% CI	*P*	*I*^*2*^	
Total	9	**1.56**	**1.27–1.92**	**<0.01**	**69.4**	**1.64**	**1.32–2.05**	**<0.01**	**55.6**	**2.24**	**1.69–2.97**	**<0.01**	**38.5**	**1.70**	**1.36–2.14**	**<0.01**	**61.8**	**1.89**	**1.23–2.90**	**0.01**	**46.3**	
HWE-yes	3	**1.05**	**0.81–1.37**	**0.71**	**0**	0.99	0.71–1.38	0.95	0	1.29	0.64–2.58	0.49	0	1.02	0.74–1.41	0.88	0	1.18	0.63–2.22	0.60	0	
HWE-no	6	**1.82**	**1.51–2.19**	**<0.01**	**54.4**	**1.94**	**1.67–2.25**	**<0.01**	**0**	**2.54**	**1.57–4.12**	**<0.01**	**48.0**	**2.04**	**1.77–2.35**	**<0.01**	**0.4**	**2.12**	**1.24–3.60**	**0.01**	**58.5**	
Ethnicity																						
Caucasian	3	1.34	0.93–1.93	0.12	67.9	**1.76**	**1.05–2.94**	**0.03**	**65.3**	1.17	0.72–1.90	0.53	0	1.69	0.98–2.90	0.06	71.5	0.95	0.61–1.49	0.84	0	
Asian	6	**1.72**	**1.37–2.15**	**<0.01**	**60.7**	**1.59**	**1.22–2.07**	**<0.01**	**58.7**	**3.15**	**2.21–4.51**	**<0.01**	**0**	**1.70**	**1.31–2.23**	**<0.01**	**63.6**	**2.73**	**1.92–3.89**	**<0.01**	**0**	
Design																						
PC	6	**1.47**	**1.10–1.96**	**0.01**	**70.5**	**1.62**	**1.15–2.28**	**0.01**	**62.2**	**1.72**	**1.18–2.49**	**<0.01**	**37.1**	**1.65**	**1.17–2.33**	**<0.01**	**66.1**	1.51	0.89–2.57	0.13	41.4	
HC	3	**1.73**	**1.28–2.34**	**<0.01**	**65.4**	**1.64**	**1.19–2.26**	**<0.01**	**58.4**	**3.24**	**2.09–5.02**	**<0.01**	**0**	**1.74**	**1.24–2.45**	**<0.01**	**66.4**	**2.80**	**1.81–4.33**	**<0.01**	**0**	
Cancer location																						
Oral	4	**1.76**	**1.36–2.27**	**<0.01**	**55.2**	**1.71**	**1.15–2.54**	**0.01**	**71.1**	**3.13**	**2.06–4.77**	**<0.01**	**0**	**1.83**	**1.26–2.66**	**<0.01**	**70.1**	**2.69**	**1.77–4.09**	**<0.01**	**0**	
Nasopharyngeal	4	**1.53**	**1.06–2.20**	**0.02**	**76.5**	**1.75**	**1.38–2.21**	**<0.01**	**5.2**	1.81	0.94–3.48	0.07	60.3	**1.74**	**1.28–2.36**	**<0.01**	**44.9**	1.52	0.79–2.90	0.21	64.2	
−819 T>C		C vs. T				TC vs. TT				CC vs. TT				TC + CC vs.TT				CC vs. TT + TC				
Total	6	**1.15**	**1.04–1.21**	**0.01**	**20.1**	1.24	0.93–1.65	0.14	65.8	**1.28**	**1.03–1.59**	**0.03**	**0**	1.27	0.99–1.64	0.06	62.0	1.19	0.98–1.45	0.08	0	
HWE-yes	4	**1.24**	**1.07–1.45**	**0.01**	**33.3**	1.33	0.75–2.37	0.33	78.3	**1.53**	**1.08–2.15**	**0.02**	**0**	1.41	0.86–2.32	0.18	73.8	1.31	0.99–1.75	0.06	0	
HWE-no	2	1.09	0.96–1.24	0.20	0	1.11	0.93–1.33	0.24	0	1.13	0.85–1.50	0.40	0	1.12	0.95–1.32	0.19	0	1.08	0.82–1.42	0.57	0	
Ethnicity																						
Asian	5	**1.16**	**1.05–1.28**	**<0.01**	**29.7**	1.27	0.94**–**1.71	0.12	72.0	**1.30**	**1.04–1.61**	**0.02**	**0**	1.30	0.99**–**1.70	0.06	68.7	1.22	0.99**–**1.51	0.07	0	
Design																						
PC	4	**1.24**	**1.07–1.45**	**0.01**	**33.3**	1.33	0.75**–**2.37	0.33	78.3	**1.53**	**1.08–2.15**	**0.02**	**0**	1.41	0.86**–**2.32	0.18	73.8	1.31	0.99**–**1.75	0.06	0	
HC	2	1.09	0.96–1.24	0.20	0	1.11	0.93**–**1.33	0.24	0	1.13	0.85**–**1.50	0.40	0	1.12	0.95**–**1.32	0.19	0	1.08	0.82**–**1.42	0.57	0	
Cancer location																						
Oral	3	1.23	0.98**–**1.54	0.08	63.6	1.44	085**–**2.44	0.18	85.5	1.6	0.97**–**1.63	0.09	2.4	1.45	0.90**–**2.35	0.13	84.3	1.17	0.91**–**1.50	0.22	0	
Nasopharyngeal	3	1.15	0.96**–**1.37	0.13	0	1.10	0.84**–**1.44	0.49	0	1.33	0.90**–**1.96	0.15	0	1.16	0.90**–**1.49	0.25	0	1.22	0.89**–**1.67	0.22	0	
**–**592 A>C		C vs. A				AC vs. AA				CC vs. AA				AC+CC vs. AA				CC vs. AA+AC				
Total	6	1.11	0.94**–**1.31	0.22	55.6	1.14	0.84**–**1.55	0.41	71.2	1.14	0.92**–**1.41	0.24	22.3	1.17	0.88**–**1.57	0.29	71.3	1.11	0.92**–**1.36	0.28	0	
HWE-yes	5	1.16	0.95**–**1.42	0.14	52.3	1.21	0.79**–**1.87	0.38	74.9	1.31	0.98**–**1.76	0.07	8.9	1.26	0.85**–**1.88	0.26	73.4	1.22	0.94**–**1.56	0.13	0	
Ethnicity																						
Asian	5	1.13	0.94**–**1.36	0.22	64.0	1.16	0.84**–**1.62	0.36	76.7	1.15	0.92**–**1.43	0.21	35.1	1.20	0.88**–**1.63	0.25	76.8	1.13	0.92**–**1.40	0.25	8.5	
Design																						
PC	4	**1.24**	**1.07–1.45**	**0.01**	**33.3**	1.33	0.75**–**2.37	0.33	78.3	**1.53**	**1.08–2.15**	**0.02**	**0**	1.41	0.86**–**2.32	0.18	73.8	1.31	0.99**–**1.75	0.06	0	
HC	2	0.95	0.84**–**1.08	0.47	0	0.94	0.79**–**1.12	0.51	0	0.93	0.70**–**1.24	0.62	0	0.94	0.80**–**1.11	0.46	0	0.95	0.73**–**1.26	0.74	0	
Cancer location																						
Oral	3	1.20	0.90**–**1.60	0.22	77.2	1.09	0.92**–**1.28	0.32	87.8	1.24	0.82**–**1.88	0.30	45.5	1.40	0.82**–**2.40	0.22	87.5	1.10	0.86**–**1.41	0.46	0	
Nasopharyngeal	3	1.03	0.87**–**1.24	0.71	16.3	0.94	0.72**–**1.23	0.65	0	1.14	0.77**–**1.69	0.50	27.8	0.99	0.77**–**1.27	0.91	0	1.14	0.83**–**1.57	0.42	25.5	

PC: Population control HC: Hospital control.

*Numbers of comparisons.

**Table 3:** Characteristics of case-control studies on IL-10 −1082A > G and −819T > C and −592A > C haplotype and head and neck cancer risk included in the meta-analysis.

Firstauthor	Year	Case	Control	Haplotype distribution								Cancer Location
				Case				Control				
				ATA	GCC	ACC	GTA	ATA	GCC	ACC	GTA	
Pratesi	2006	89	130	46	79	53	0	66	110	84	0	Nasopharyngeal
Wei	2007	198	210	235	84	69	8	278	51	86	5	Nasopharyngeal
Yao	2008	280	300	319	106	104	31	377	73	125	25	Oral
Hsu	2015	145	112	167	16	107	0	157	16	51	0	Oral

**Table 4:** Meta-analysis of the IL-10 −1082A > G, −819T > C and −592A > C haplotype and head and neck cancer risk.

Contrast		OR	95% CI	*P*	*I*^2^	*P*_Egger’s test_
GCC vs. ATA	Total	**1.44**	**1.04–2.00**	**0.03**	**52.6**	0.21
	Caucasian	1.37	0.83–2.25	0.22	66.3	
	Asian	1.45	0.72–2.92	0.30	66.7	
	Oral	1.39	0.79–2.44	0.25	54.1	
	Nasopharyngeal	1.44	0.77–2.68	0.25	75.9	
ACC vs. ATA	Total	1.14	0.80–1.61	0.47	69.9	0.83
	Caucasian	0.96	0.74–1.25	0.77	0	
	Asian	1.36	0.66–2.79	0.40	85.9	
	Oral	1.37	0.70–2.72	0.36	86.6	
	Nasopharyngeal	0.93	0.70–1.25	0.65	0	
GTA vs. ATA	Total	1.54	0.94–2.52	0.09	0	NA

NA: not available.
